# Dental Calculus as a Potential Biosource for Human Papillomavirus Detection in Oral Squamous Cell Carcinoma

**DOI:** 10.31557/APJCP.2020.21.10.3093

**Published:** 2020-10

**Authors:** Natallia Pranata, Ani Melani Maskoen, Edhyana Sahiratmadja, Sunardhi Widyaputra

**Affiliations:** 1 *Graduate School of Biomedical Sciences, Master Program, Faculty of Medicine, Universitas Padjadjaran, Bandung, West Java, Indonesia. *; 2 *Department of Oral Biology, Faculty of Dentistry, Maranatha Christian University, Bandung, West Java, Indonesia. *; 3 *Department of Oral Biology, Faculty of Dentistry, Universitas Padjadjaran, Jatinangor, West Java, Indonesia. *; 4 *Department of Biomedical Sciences, Faculty of Medicine, Universitas Padjadjaran, Jatinangor, West Java, Indonesia.*

**Keywords:** Dental informatics, bioinformatics, microbial genetics, oral carcinogenesis, oral pathology

## Abstract

**Objective:**

The infection of human papillomaviruses (HPVs) plays a role in the development of oral squamous cell carcinoma (OSCC). A poor oral hygiene and dental calculus may cause the infection to persist. Therefore, this study aimed to assess whether this dental calculus could serve as a potential biosource in early detection of HPVs in patients with OSCC.

**Methods:**

DNA was isolated from the dental calculus of people diagnosed with OSCC, and MY09/11 primer set was used to detect the presence of HPV. The positive samples were further sequenced and aligned using megablast NCBI BLAST tool to identify the HPV genotype.

**Results:**

Electrophoresis examination showed that 4 of 14 samples collected (29%) had a clear single band, of which three had 97% to 99% similarity to a high-risk genotype HPV-58. Meanwhile, the other sample had 99% similarity to an unclassified papillomaviridae.

**Conclusion:**

Dental calculus is a promising source of HPV in oral cavity and could be used as a biomarker for early detection.

## Introduction

Oral cancer is often diagnosed at a late stage, and have a very low 5-year survival rate (Dissanayaka et al., 2012; Kintawati and Pramesti, 2019). The global incidence of lip, oral cavity, and pharynx malignancies is predicted to increase to 62% by the year 2035, (Shield et al., 2017) and 99% is diagnoses as oral squamous cell carcinoma (OSCC) (Rivera and Venegas, 2014). The OSCC is considered to be a preventable disease that is amenable to early detection and treatment. 

The cause of OSCC is multifactorial, one of which is human papillomavirus (HPV) infection. Previous studies showed that 73% to 87% incidence of OSCC is caused by HPV (Gillison et al., 2015; Goot-Heah et al., 2012; Kim., 2016). However, recent epidemiologic evidence suggested the infection may also be an independent risk factor (Turner et al., 2011). Therefore, the carcinoma is classified based on the presence of HPV. Also, those with OSCC with positive HPV has a better prognosis. The E6 protein from this virus inhibits p53 activation, and the TP53 in OSCC with positive HPV is very rare in mutation (Rampias et al., 2014). Also, not all types of this virus have the same potential involvement in carcinogenesis. Based on its role, it is classified into high (HR) and low risk (LR) groups.

HPV is latent, which means it does not develop immediately after infecting the cell. Therefore, a biological source that is able to keep the virus in the oral cavity for a long period is needed (Gillison et al., 2015). The presence of HPV could be used as a biomarker for early detection of OSCC.

Several methods are being developed for the detection of this virus such as the molecular techniques that are based on genomic probe technology, which are the gold standard. The polymerase chain reaction (PCR) for amplification of its DNA are highly sensitive, specific, and widely used in many clinical and epidemiological studies (Abreu et al., 2012). Also, a stretch of highly conserved amino acid residues was contained in a 291 bp segment of L1 ORF that is spanned by MY09 and MY11 primers (de Villiers, 2013). This primer set has greater sensitivity for detecting HPV.

Currently, the non-invasive biological sources for detecting this virus are saliva and exfoliated cells (Leemans et al., 2011; Wimardhani et al., 2015). This is because saliva has abundant genomic and proteomic content (Ramseier et al., 2009), which is useful in diagnostic test. The limitation of this option is that saliva can only provide a present overview, whereas for the carcinogenesis process, continuous presence of HPV for long period is more important. Since the pathogenesis of OSCC is quite unique, another alternative of early diagnostic sources is needed. 

In the oral cavity, dental calculus, which is a plaque calcification is found around the teeth and gingiva. Meanwhile, supragingival calculus is formed from saliva precipitate, and those from gingival crevicular fluid (GCF) (Weyrich et al., 2015). During biomineral maturation process, several biological contents around the oral region should be trapped, including the exfoliated virus contained cells. Also, DNA, protein, and amounts of dietary biomolecules have been successfully extracted from ancient dental calculus (Warinner et al., 2014). It can serve as a good biological data storage and a potential source for detecting the molecular marker of the latent pathogen that causes oral disease (Metcalf et al., 2014)

Therefore, this study aimed to detect HPV in the dental calculus of OSCC patients. The presence of this virus would intrigue a novel non-invasive early detection of OSCC.

## Materials and Methods

*Study samples*

Patients diagnosed with OSCC in the Oncology Subdivision Department of General Surgery Hasan Sadikin General Hospital, Bandung, were included from May to October 2017. The diagnosis of OSCC was histopathologically confirmed by the pathologist. After consent, dental calculus was obtained from all the participants. Data on gender, age, dental scaling record and smoking habits were also confidentially noted. 

Meanwhile, this protocol was approved by the Health Research Ethics Committee Faculty of Medicine Universitas Padjadjaran, Bandung, Indonesia (no. 0317030299).

*Sample collection*

Supra- and subgingival dental calculus was obtained using electric scaler (DTE^®^ D1, Woodpecker, Guilin, China) without being invasive. 

*DNA isolation*

The obtained sample was transferred into innuSPEED Lysis Tube J (Analytic Jena, Jena, Germany) and added with 1 mL EDTA 0,1 M., then pulverised into a homogenous suspension using speedMill Plus (Analytic Jena, Jena, Germany). This suspension was transferred into a sterile 1.5 ml microcentrifuge tube, and incubated at room temperature (23-25°C) for 1 hour. It was then mixed vigorously with pulsed vortexing for 15 sec every 15 min. This decalcification method is the most suitable for optimal DNA concentration and purity based on preliminary study (submitted data). Subsequently, the calcium was precipitated by centrifugation at 1,000 rpm for 5 mins. The supernatant was then transferred into a new tube. Also, the cell and organic material were precipitated by centrifugation at 12,000 rpm for 10 min, followed by removing the supernatant. The precipitate was then prepared for DNA isolation. 

DNA was isolated from the cell precipitation of dental calculus with CLART^®^ Human Papillomavirus 2 Extraction and Purification Kit (Genomica, Madrid, Spain), using the procedure recommended by the manufacturer. It was resuspended in a 50 μl elution buffer. Meanwhile, its concentration and purity were measured using Maestro nano spectrophotometer (MaestroGen, Hsinchu, Taiwan). Therefore, to verify the efficiency of the extraction process, human gene Cystic Fibrosis Transmembrane Conductance Regulator (CFTR) primers were used. 

*Amplification with MY09 and MY11 primer set*

The presence of HPV DNA was determined by Mastercycler gradient thermocycler (Eppendorf, Germany) with MY09 and MY11 primer set. Also, the amplification assays were conducted in a 25 μl volume mixture containing: 12,5 μl of PCR buffer (KAPABiosystems, Massachusetts, US) 1,25 μl of each primer, 7,5 μl of nuclease-free water and 2,5 μl of DNA sample. The thermal cycling conditions involved initial denaturing for 3 min at 95°C, followed by 37 amplification cycles for 30 sec at 95°C, 15 sec at 48°C and 30 sec at 72°C; Also the final extension step lasted 5 min at 72°C. Positive and negative controls were used in each assay to assess whether the DNA was contaminated. Furthermore, PCR amplification using generic primers was conducted twice, at different times, to rule out sample contamination and reduce the number of false positives (Camargo et al. 2011). The positive control, containing HPV type 58 DNA extracted from a cervical cancer patient, was used for each reaction. Also, the obtained 5µl of PCR products were run on 1% agarose gels stained with pegGreen (VWR Company, Ohio, USA) and then visualised on an ultraviolet transilluminator.

*HPV genome sequencing*

The PCR products that have visualized band at 450 bp were sequenced via submission to First BASE Laboratories Sdn. Bhd, which used Applied Biosystems™ Genetic Analyzer with Sanger sequencing method. The query sequences were compared to the databases using the NCBI BLAST tool (Johnson et al., 2008). Futhermore, the selected programs were megablast (highly similar sequences), which were used to identify intraspecies and blastn to align with HPV type 16 and 18.

## Results

In total, there were 14 participants consisting of 7 male and 7 females. Based on dental scaling record, 64% have not had scaling treatment in the last 6 months, while 36% have. All the participants have smoking habits, 43% are active, while 57% are passive. The collected clinical data are shown in Table 1.

**Figure 1:**
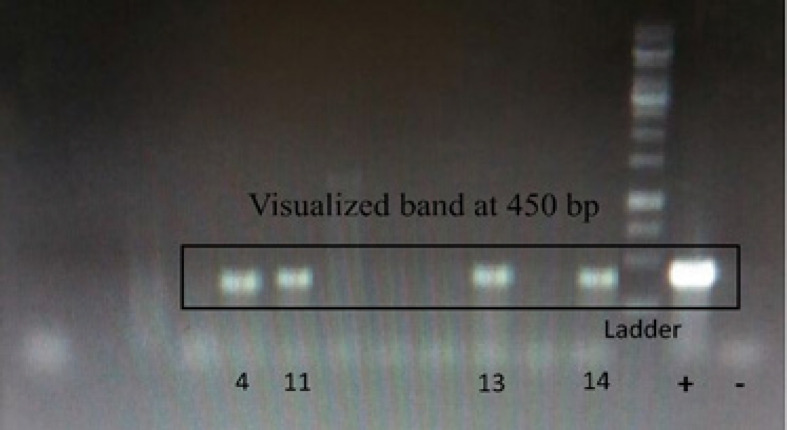
Gel electrophoresis of PCR products with MY09 and MY11 primer set. Visualized band at 450 bp; Lane + is positive sample for HPV-58; Lane – is negative sample. Numbers indicate sample no

Also, PCR analysis was performed on all the samples. 4 samples showed visualized band at 450 bp, as presented in Figure 1. Meanwhile, the HPV positive was 29% (4 of 14) (data no shown). Two positive samples were male, never had dental scaling treatment, and active smokers. The rest were females, never had the treatment, and passive smokers. 

Positive query sequences were compared with the databases using the NCBI BLAST tool. The selected program was highly similar (megablast). Also, the highest number was 3 samples for HPV type 58 with 97% to 99% similarity. This means 75% (3 of 4) of all the positive samples. The rest were identified as unclassified Papillomaviridae (similarity 99%). This query sequences can also be aligned with the database of type 16 and 18 using the blastn program. All the query sequences had 66% to 79% similarity with HPV type 16 and 18 (Table 2).

**Table 1:** The Clinical Characteristics of Oral Squamous Cell Carcinoma Patients (N=14) from May to October 2017

Gender	Age	n	Dental scaling record		Smoking habits	
			Never	Last six months	Active	Passive
Male	30-39	3	3*		3*	
	50-59	2	1	1	1	1
	60-69	2	2*		2*	
Female	20-29	3	1	2		3
	50-59	4	2**	2		4**
Total	n (%)	14	9 (% 64)	5 (% 36)	6 (% 43)	8 (% 57)

Note : *, 1 sample of this clinical characteristics category is shown HPV positive; **, 2 samples of this clinical characteristics category are shown HPV positive

**Table 2:** The HPV Positive Query Sequences Against the BLAST Sequence Databases

Sample no.	Megablast program		Blastn program Similarity	
	HPV type	Similarity	HPV 16	HPV 18
11	HPV type 58	99%	79%	79%
13	HPV type 58	97%	79%	79%
14	HPV type 58	99%	79%	79%
4	Unclassified Papillomaviridae	99%	66%	66%

## Discussion

Dental calculus is common in majority of adults worldwide in both healthy and cancer patients, and is composed primarily of calcium phosphate salts. Its formation is population specific and is affected by oral hygiene, access to professional care, diet, age, ethnicity, time since last dental cleaning, systemic disease, and the use of medications. Therefore, this calculus is a good biological data storage and a useful source for detecting molecular marker of the latent pathogen (Metcalf et al., 2014), including entrapped oral desquamated cells. Also, it can entrap virus, both from the cells and saliva, including HPV as one of the predicted causes of OSCC. 

This virus has a unique and complex lifecycle with a tropism for epithelia. In general, the virions invade through damaged areas of the epithelium and infect the basal cells. It is postulated that they initially attach to the heparan sulfate proteoglycan (HSPG) on the basal membrane, migrate to keratinocyte receptor, and then enter the cells. At the basal cells, HPV suppresses cell replication to a “maintenance level” or “latent infection mode.” Furthermore, it preserves the DNA synthesis potential of the infected cells to maintain viral genome replication. Therefore, at the end of differentiation, a tremendous level of genome amplification and late gene expression takes place. after the virions assemble, they are then released into the oral cavity with desquamated cells (Kajitani et al., 2012). 

In this study, PCR analysis of 4 samples showed visualized band at 450 bp (Figure 1), with 29% HPV (4 of 14) (Table 1). When the positive query sequences were compared with the databases using NCBI BLAST tool, the highest number was 3 samples for type 58 with 97% to 99% similarity. Recent epidemiologic evidence suggested that persistence of infections by HR HPV types is the single greatest risk factor for cancer progression (Bodily and Laimins, 2011; Turner et al., 2011). Also, cancers arise in persistent lesions through the action of two viral oncoproteins, E6 and E7, which play key roles in maintaining the infections. Only the HR HPV E6 and E7 contribute to the development of malignancies. The E6 and E7 proteins modulate the activities of several cellular factors that control viral lifecycle, and mediate the development of cancers (Bodily and Laimins, 2011). 

Most of the positive samples in this study were identified as type 58 with 97% to 99% similarity (Table 2). Also, type 58 is classified a high risk for malignant progression (Panigoro et al., 2013; Bzhalava et al, 2015). This type is reported as the most common genotypes found in cervical cancers after HPV 16 and 18 in Eastern Asia and in Thailand (Khunamornpong et al. 2016). These infections are uncommon in Chinese OSCC patients (Chen et al, 2016). Also, there are very few research on HPV 58 in the oral cavity, especially in Indonesia.

Previous studies found that high risk HPV 16 and 18 are prevalent in OSCC patients (Gan et al., 2014; Goot-Heah et al., 2012; Sasahira et al, 2014). Meanwhile, this study performed a query sequence from the positive samples and aligned with the type 16 and 18 databases. All the sequences have 66% to 79% similarity with type 16 and 18 (Table 2), based on the nucleotide sequence of ORF coding for the capsid protein L1. These types are phylogenetically clustered within the species groups of Alpha papillomavirus 9 (HPV16-related) or 7 (HPV18-related) (Chen et al., 2013). This means the samples of this study belong to the same genus, and also have similar biological and medical properties with HPV 16 and 18.

The oral cavity contains a wide spectrum of the virus types and little is known about the existence of novel types (Bottalico et al., 2011; Martin et al., 2014). Interestingly, this study showed the rest of the positive samples were identified as unclassified Papillomaviridae with 99% similarity (Tabel 2). Meanwhile, further study on this novel unclassified HPV type is in progress. 

In conclusion, the presence of HPV in dental calculus is a potential source for identifying latent pathogens in pathogenesis studies of oral cancer, especially OSCC.
